# Reporting in Experimental Philosophy: Current Standards and Recommendations for Future Practice

**DOI:** 10.1007/s13164-018-0414-3

**Published:** 2018-07-29

**Authors:** Andrea Polonioli, Mariana Vega-Mendoza, Brittany Blankinship, David Carmel

**Affiliations:** 1 Department of Philosophy, University of Birmingham, 3 Elms Road, Edgbaston, Birmingham, B15 2TT UK; 2 Department of Psychology, University of Edinburgh, Edinburgh, UK; 3 School of Psychology, Victoria University of Wellington, Wellington, New Zealand

## Abstract

Recent replication crises in psychology and other fields have led to intense reflection about the validity of common research practices. Much of this reflection has focussed on reporting standards, and how they may be related to the questionable research practices that could underlie a high proportion of irreproducible findings in the published record. As a developing field, it is particularly important for Experimental Philosophy to avoid some of the pitfalls that have beset other disciplines. To this end, here we provide a detailed, comprehensive assessment of current reporting practices in Experimental Philosophy. We focus on the quality of statistical reporting and the disclosure of information about study methodology. We assess all the articles using quantitative methods (*n* = 134) that were published over the years 2013–2016 in 29 leading philosophy journals. We find that null hypothesis significance testing is the prevalent statistical practice in Experimental Philosophy, although relying solely on this approach has been criticised in the psychological literature. To augment this approach, various additional measures have become commonplace in other fields, but we find that Experimental Philosophy has adopted these only partially: 53% of the papers report an effect size, 28% confidence intervals, 1% examined prospective statistical power and 5% report observed statistical power. Importantly, we find no direct relation between an article’s reporting quality and its impact (numbers of citations). We conclude with recommendations for authors, reviewers and editors in Experimental Philosophy, to facilitate making research statistically-transparent and reproducible.

## Introduction

Philosophers have recently started to adopt empirical methods to address research questions of philosophical relevance. This practice is often referred to as Experimental Philosophy (Knobe and Nichols 2008; Alexander 2012; Knobe et al. 2012; Machery and O’Neill 2014; Sytsma and Buckwalter 2016), although it incorporates both experimental and correlational studies. More generally, it seems that what best characterizes this recent trend is an attempt to employ quantitative methods to make progress in philosophy (Knobe 2015).

The application of quantitative methods raises a number of important issues for this field. Previous research has discussed a constellation of ethical issues that have arisen since philosophers started to conduct empirical research (Polonioli 2017). Yet experimental philosophers should also be concerned with ongoing discussions, in several empirical fields, about whether common scientific practices in design, analysis, and reporting ought to be revised (Begley and Ellis 2012; Ioannidis 2005; Miguel et al. 2014; Simmons et al. 2011).

Such discussions are particularly heated in psychology, where a substantial number of findings have recently failed to replicate (Makel et al. 2012; Maxwell et al. 2015; Open Science Collaboration 2012, 2015; Pashler and Harris 2012; Simons 2014). The ‘crisis of confidence’ within psychology (Pashler and Wagenmakers 2012) arose from finding that many published results could not be replicated when competent independent researchers executed high-powered replication attempts that duplicated the original methodology as closely as possible. For example, the Reproducibility Project was set up as an open large-scale attempt at estimating the replicability of psychological science (Open Science Collaboration 2012); it attempted to replicate 100 studies selected from 2008 issues of three leading psychology journals (*Psychological Science*, *Journal of Personality and Social Psychology*, and *Journal of Experimental Psychology: Learning, Memory, and Cognition*)*.* The results of this effort, published a few years later (Open Science Collaboration 2015) revealed that only 36% of the replications had statistically significant results, as opposed to 97% of the original studies; and effects in replications had, on average, half the magnitude of those originally reported.

The problem does not seem to be limited to psychology: replication projects in medicine (Prinz et al. 2011) and behavioral economics (Camerer et al. 2016) have also delivered relatively low success rates. Replication crises are arguably multifaceted and many different factors are likely to contribute to low reproducibility rates. Individual misconduct or even outright fraud are known to occur, but are likely to be the exception rather than the rule and cannot account for the problem (Fanelli 2009). Other possible sources of irreproducibility include factors that arise at various stages of the research process: design (e.g., selection biases), analysis (e.g., questionable research practices such as p-hacking) and publication (e.g., a preference for the publication of statistically significant findings) (Nosek et al. 2015; Ioannidis 2005, 2014; Ioannidis et al. 2014; Fanelli 2010; Simmons et al. 2011; John et al. 2012). In Psychology, at least, increased awareness of these sources of poor reproducibility has led to recent changes, including the large-scale adoption of several new practices in analysing and reporting research, which give reason for optimism (Nelson et al. 2018). These desirable practices—described in further detail below—are highly relevant to Experimental Philosophy research, which has mostly attempted to apply psychological methods in tackling philosophical questions. Notably, as a field, Experimental Philosophy seems aware that reproducibility can and should be monitored, with both organized replication projects (Cova et al. 2018) and online resources tracking replicability (http://experimental-philosophy.yale.edu/xphipage/Experimental%20Philosophy-Replications.html).

Critiques of research practices and the editorial handling of research outputs are not a recent phenomenon. For instance, within psychology Gigerenzer (2004) argued that statistical inference is an incoherent hybrid of the ideas of Fisher and of Neyman and Pearson. Others have drawn attention to the impact of publication biases on the literature, namely the publication or non-publication of research findings depending on the nature and direction of the results. Whilst publication biases seem to be common in many fields (Ioannidis 2005; Ioannidis et al. 2011), psychology is one of the fields where their impact has generated most discussion recently (Ferguson and Brannick 2012; Francis 2012, 2015; Francis et al. 2014; Ioannidis 2012). But again, this is not new: Rosenthal (1979) pointed out almost four decades ago that the preference for publishing positive (statistically significant) results is conducive to a file-drawer effect, resulting in an over-representation of false positives in the published record. The recent replication crisis has led an increasing number of scholars to issue warnings about the shortcomings of common practices. Consequently, it has become easier—though still by no means easy—to publish non-significant results (at least in psychology) and initiatives such as Open Science Framework^1^ have promoted greater transparency of research by encouraging openness of data and allowing pre-registration of studies.

Importantly, consensus has also emerged that current reporting practices are problematic because insufficient details are often provided, preventing accurate interpretation and evaluation of findings (Miguel et al. 2014; Simmons et al. 2011).

Some researchers have also stressed that statistical standards need to be revised. For instance, Benjamin et al. (2018) suggested a stricter threshold for defining statistical significance, whilst others stress instead that *p* values should not be treated as reliable measures given that they might vary (sometimes dramatically) across replications, even when the effects are real (Halsey et al. 2015; see also Cumming 2008). Moreover, some have argued that null-hypothesis significance testing (NHST) and Bayesian inference may lead researchers to draw different conclusions in certain cases, and that the use of Bayesian statistics should be preferred when possible (Dienes and McLatchie 2018; Wagenmakers et al. 2018).

The concerns regarding statistical analysis and reporting, reviewed above, have led to various suggestions regarding the kinds of analyses that should be done and reported in order to improve the reproducibility of findings. These suggestions include a greater focus on full reporting of descriptive statistics, the use of confidence intervals and effect sizes, and the employment of power calculations. A growing body of literature provides details on the justification for these suggestions and how they should be implemented practically (Tellez et al. 2015; Fritz et al. 2012; Tressoldi and Giofré 2015), discussing for instance which type of effect size to use in different circumstances (Sullivan and Feinn 2012; Lakens 2013).

These views and principles have become mainstream in psychological research. The 6th edition of the *American Psychological Association Publication Manual* greatly emphasizes the importance of reporting elements such as effect sizes, confidence intervals, and extensive description of procedures, which help convey the most complete meaning of the results (2010, p. 33). In addition, from January 2014 *Psychological Science,* the flagship journal of the Association for Psychological Science, recommends the use of the “new statistics”—meta-analyses, effect sizes and confidence intervals—to avoid problems associated with null-hypothesis significance testing^2^ (notably, confidence intervals are directly related to *p*-values—see, for example, Altman and Bland 2011—so the recommendation to replace one with the other is controversial; however, it is a testament to the current goal, within experimental psychology, of searching for more robust ways to find and report results). *Psychological Science* also encourages complete reporting of study design and methods. To allow authors to provide clear, complete, self-contained descriptions of their studies, the Methods and Results sections no longer count towards the total word limit. Overall, there is recent consensus in the literature around the following two recommendations:

- Estimation based on effect sizes, confidence intervals, and meta-analysis usually provides a more informative analysis of empirical results than does null-hypothesis significance testing alone.
- Transparency in the methodologies and procedures used to obtain research findings is required to allow for replication of such findings.

In other words, a reader should be able to assess the merit of a study’s findings through a full, statistically-transparent set of reported results, and identify the conditions necessary to conduct a replication (either exact or conceptual) of the original research design. Notably, the APA manual from 2010 and the Psychological Science submission guidelines from 2014 emphasize these aspects of reporting, but the need to fulfil these requirements has been recognized for decades; the problem was not that researchers were unaware of that it is better to report full methods and statistical measures like effect sizes (e.g., Cohen 1994), but that in practice, they have not tended to do so. We know this because several studies have assessed reporting standards over the years, generally finding a need for improvement: Matthews et al. (2008) analyzed 101 articles published between 1996 and 2005 in 5 education journals, and found that the proportion of articles that reported effect sizes in their results increased from an average of 26% across journals in 1996–2000 to 46% in 2001–2005. As Matthews et al. (2008) noted, this shows a gradual improvement but the numbers were still low (and did not exceed 60% in any single journal), despite the fact that the APA manual already called for reporting effect sizes during the surveyed period. In line with this, Sun et al. (2010) surveyed articles published between 2005 and 2007 in six American Psychological Association (APA) journals, and Fritz et al. (2012) examined articles published a few years later (2009–2010) in a leading psychology journal (Journal of Experimental Psychology: General); both investigations found that only around 40% of surveyed articles reported effect sizes. A significant change—at least in psychology—seems to have come about, however, in the 2010s, possibly following the field’s replication crisis (Nelson et al. 2018). For example, Tressoldi et al. (2013) examined papers published in 2011 in four high-impact and three field-specific psychology journals, and found that in most journals (the notable exceptions were Nature and Science) effect sizes and/or confidence intervals were reported in a majority of articles—in the Journal of Experimental Psychology: Applied, for example, these measures were included in 90% of papers. Corroborating this trend, Counsell and Harlow (2017) recently reported that over 90% of papers published in four Canadian Psychology journals in 2013 reported measures of effect size, suggesting that the constant calls for reporting effect sizes appear to have had an effect.

However, reporting practices may differ across research fields, as well as across the hierarchy of publication venues. In particular, as noted in the previous paragraph, there has been criticism of the sparse reporting standards imposed by some high-visibility, high-impact journals: Tressoldi et al. (2013) documented widespread use of null hypothesis significance testing without any use of confidence intervals, effect size, prospective power and model estimation in high-impact journals such as *Science* and *Nature*.

In addition, a number of studies—including recent ones—have documented that researchers often fail to provide sufficient methods information for conducting replications (e.g., Sifers et al. 2002; Raad et al. 2007; Bouwmeester et al. 2012; Pierce et al. 2014). For instance, Sifers et al. (2002) explored reporting of demographic and methodological information in four major paediatric and child psychology journals. They found that information about sample size and age was almost always reported, yet providing details about ethnicity, socioeconomic status (SES) and the exclusion/inclusion of participants was far from being the norm. More recently, Pierce et al. (2014) also found that the reporting of ethnicity information in three major autism research journals was largely unsatisfactory.

All the above is of particular relevance to Experimental Philosophy, a new field in the process of establishing its methodologies and reporting conventions. Furthermore, Experimental Philosophy is interested in people’s attitudes and behaviors, and largely employs methods and analysis strategies commonly used in Experimental Psychology, making it a sub-field, or at least sister-field, of that older discipline. It would thus be advisable for Experimental Philosophers to take heed of both established experimental design principles (Carmel 2011) and the recent turmoil regarding analysis and reporting practices that science in general, and Psychology in particular, have been undergoing. The need to be vigilant in trying to avoid other fields’ past mistakes is particularly important because Experimental Philosophy research is conducted mostly by philosophers, who are often not trained in experimental work and only rarely receive any training in statistics. To their credit, most Experimental Philosophers (to the best of our knowledge) do make an effort to acquire some statistical expertise, and many collaborate with trained psychologists. We also acknowledge that experimental philosophers are a heterogeneous group, and that some have gained considerable quantitative competence by informal means. But does the publication record in Experimental Philosophy demonstrate that these efforts are sufficient?

The present investigation aims to contribute to the literature on reporting practices in scientific research and to the healthy development of Experimental Philosophy, by empirically exploring the reporting of methods, analyses, and results in Experimental Philosophy publications. Assessing the overall quality of research in a field is complicated and may be impossible; there is no consensus, for example, on how to quantify the quality of research designs, so opinions may (and frequently do) differ on this aspect of any given study. But—as the literature cited above indicates—many of the proposed solutions for poor replicability in empirical research emphasize the consistent adoption of appropriate reporting practices. Use of these practices—specifically, the reporting of sufficient method information that would allow others to replicate the study, and the use of statistical measures such as effect sizes and confidence intervals to complement *p*-values—can be quantified; furthermore, at least in Psychology, improvements in reporting standards (e.g., Counsell and Harlow 2017) have occurred alongside an overall shift in a host of practices that has led to optimism about resolving the field’s replication crisis (Nelson et al. 2018). Although it is too soon to tell whether psychological findings have become more reproducible in recent years, and whether any such improvements can be causally linked to changes in reporting practices, it is not too soon to examine whether Experimental Philosophy has taken heed of these developments and adopted appropriate reporting standards. Therefore, in the present study we focus on experimental philosophers’ reporting of effect sizes, confidence intervals and statistical power, as well as the transparency of information about research procedures (see full details in the Method and Supplementary Information (https://osf.io/yp2kg)). In doing so, we hope to help the Experimental Philosophy community establish appropriate reporting standards and ensure that, over time, the body of work produced in this field is largely reproducible.

## Methods

We examined design, analysis, and reporting of research in Experimental Philosophy. We defined Experimental Philosophy broadly (Rose and Danks 2013; Machery and O’Neill 2014) and identified relevant Experimental Philosophy papers by following a modified version of Polonioli’s (2017) methodology.

### Inclusion Criteria

First, we selected a broad sample of peer-reviewed philosophy journals. A natural way to identify the most important journals is by appealing to the journal impact factor (IF), which is the most common measure of a journal’s impact and quality (though see criticisms of this measure by Horvat et al. 2016; Brembs et al. 2013; Moustafa 2014). Unfortunately, an IF is unavailable for most journals in philosophy; other available classifications of journals were thus considered in our study (Polonioli 2016). One quantitative ranking is provided by the h-index, and it is possible to find a ranking of philosophy journals based on this last metric.^3^ Informal polls are also a popular way of ranking philosophy journals, and a rather established ranking is published on the blog *www.leiterreports.com*. Here, we considered all of the journals that both publish original peer-reviewed research and are included in rankings based on h-index and Leiter Report’s poll, and selected the top-20 relevant journals from each of these two rankings. Of the journals included in either ranking, only the journal *Philosophy Compass* was excluded because it publishes only (typically invited) review articles. Because of the partial overlap between the lists, the sample eventually included the 29 journals.

Second, we selected all papers employing quantitative methods that were published between 2013 and 2016 in the 29 philosophy journals above. Polonioli (2017) considered only 3 years (2013–2015); here we considered an additional, fourth year. Further, unlike Polonioli (2017), we excluded qualitative research articles because in the current study the focus is on the handling of quantitative results. To select the relevant papers, we accessed the PDF version of each article published in the chosen journals between 2013 and 2016, and searched for the keywords ‘experiment,’ ‘empirical,’ ‘subject(s),’ ‘participant(s),’ ‘sample,’ ‘test,’ and ‘statistic(al).’ In cases where we deemed the keyword-based search strategy to be less effective for discriminating between empirical research and literature reviews, we read the paper. This process resulted in the identification of 134 articles as quantitative research articles. Information regarding the journals in our sample and the number of quantitative articles per journal is listed below (for a full list of the articles, see the Supplementary Information (https://osf.io/yp2kg)):

1. *Noûs (n = 4)*
2. *Philosophical studies (n = 11)*
3. *Philosophy and Phenomenological Research (n = 4)*
4. *Mind (n = 1)*
5. *Analysis (n = 2)*
6. *Synthese (n = 13)*
7. *Mind and language (n = 7)*
8. *Philosophers’ Imprint (n = 3)*
9. *Australasian journal of philosophy (n = 2)*
10. *Erkenntnis (n = 2)*
11. *Review of Philosophy and Psychology (n = 25)*
12. *Ergo (n = 3)*
13. *Philosophical Review (n = 0)*
14. *Philosophical Quarterly (n = 0)*
15. *Canadian Journal of Philosophy (n = 0)*
16. *Philosophical Psychology (n = 44)*
17. *Ethics (n = 0)*
18. *Journal of Philosophy (n = 0)*
19. *Phenomenology and the Cognitive Sciences (n = 0)*
20. *Journal of Consciousness Studies (n = 11)*
21. *Philosophical Perspectives (n = 0)*
22. *Ratio (n = 2)*
23. *Journal of Philosophical Logic (n = 0)*
24. *Pacific Philosophical Quarterly (n = 0)*
25. *American Philosophical Quarterly (n = 0)*
26. *Studies in Philosophy and Education (n = 0)*
27. *European Journal of Political Theory (n = 0)*
28. *Proceedings of the Aristotelian Society (n = 0)*
29. *European Journal of Philosophy (n = 0)*

### Procedure

The selected articles were screened according to an adapted and expanded version of the list of categories used by Tressoldi et al. (2013). We searched for reporting of confidence intervals as well as measures of effect size, interpretations of reported effect size and details on prospective statistical power. Presence of these items was coded in a binary fashion (present/absent). Following Tressoldi et al. (2013), we applied lenient criteria—a feature was coded as present if at least one instance of it appeared in the paper (i.e., if a single effect size was reported in a paper, we coded effect sizes as present in that paper, even if there were other points in that paper where an effect size could have been reported but was not). We also screened papers to identify mentions of *p* values or null hypothesis significance testing, and the use of error bars in figures. Further, we also explored the extent to which papers adopted Bayesian statistics rather than null hypothesis significance testing. For the full coding instructions we worked with, see the Supplementary Information (https://osf.io/yp2kg).

The procedures and methodology used in our study also try to complement the perspective offered by Tressoldi et al. (2013) by exploring further aspects of reporting. These include reporting of information on sample size and demographics, details of study design, and criteria for exclusion of participants. Finally, we examine reports of prospective and/or observed statistical power. By covering a wider set of aspects than previous efforts, the present study is better equipped to provide a snapshot of current practices with regard to design, analysis, and reporting of research in Experimental Philosophy. It allows for a health check of practices in the field, informing discussions about findings’ replicability as well as the possibility of their inclusion in meta-analytic studies.

We also examine the relationship between citations and quality of reporting. The association between reporting standards and impact (in the form of citations) is currently unclear. On the one hand, publications may be cited for a variety of reasons. For instance, researchers may cite others to support their own claims, methodology or findings. Other papers are cited in order to criticize their central claims (Harwood 2008). Some papers are cited as examples of well-conducted research, while others might be cited as examples of research that is poorly designed or conducted (Aksnes and Sivertsen 2004). Nevertheless, while citation counts are a function of many variables, when a particular paper is cited more than others it is usually assumed that this reflects its higher quality (Bornmann et al. 2012); even when authors disagree with cited research, it is assumed they would not go to the trouble to argue with low-quality work, and that—at least in the long run—low-quality work will be condemned to oblivion while good work continues to get cited (and sometimes debated). This assumption underlies the importance, in present-day academia, of individual researchers’ h-index and the impact factors of the journals they publish in: both citation measures influence careers by direct effects on promotions, tenure decisions, and success in research funding applications (Acuna et al. 2012).

But is the assumed association between quality and number of citations real? Egghe and Rousseau (1990) suggested that four important assumptions form the basis for all reliance on citation counts. First, citing an article implies actual use of that document by the citing author; second, citation reflects the merit (quality, significance, impact) of the cited article; third, the best possible work relevant to any point is cited; and fourth, a cited article is related in content to the one that cites it. In light of these assumptions, it is important to distinguish between the normative claim that quality *should* be a key factor in determining citations and the empirical claim that quality *does* correlate with citation counts. For example, Baird and Oppenheim (1994) investigated citations in Information Science, and concluded that “citation counts mean a statistical likelihood of high quality research”. Conversely, Nieminen et al. (2006) examined citations of studies in Psychiatry—specifically to determine whether reporting quality and statistical analyses were associated with citations—and found no such correlation. Even if we disregard the normative question of whether reporting quality should be a factor in deciding whether a given paper gets cited, it is useful to examine whether in practice, the reporting quality of studies in Experimental Philosophy is reflected in their impact as indexed by number of citations. We thus examined the number of times each of the analyzed articles has been cited^4^ and explored whether the citation count was related to our measures of reporting quality.

Finally, we also explored whether having at least one non-philosopher among the authors was associated with a higher quality of reporting. We considered the affiliations listed on the published article and in cases in which authors were affiliated to an interdisciplinary center or no clear information about their institution was available, the subject of their PhD was considered to determine the relevant subject area.

We used point-biserial correlations to assess the relationship between the number of citations and various dimensions of reporting quality. We also used Chi-square tests of independence, where appropriate, to explore the association between authors’ affiliation and relevant variables pertaining to reporting quality. Statistical analyses were performed using SPSS v. 22, JASP 0.8.3.1 (JASP Team 2016), and R statistical software (R Development Core Team 2018).

### Inter-Coder Reliability

To ensure that idiosyncratic biases and coding errors did not affect the assessment of empirical papers, articles were coded independently by three coders (authors AP, MVM and BB). Seventy-two papers were coded by at least two different authors; for these, as Table 1 shows, percentage of agreement ranged from 96% to 100% on all variables, with Kappa coefficients ranging from .66 to 1 (all *p*s < .001).

**Table 1:** Summary of agreement measures for the papers with two coders

Variable	Frequency agreement (out of *72)*	% Agreement	*k*	Asymp. S. E.	95% BCa CI
Author characteristics					
First author a philosopher	70	97	0.911	0.062	[0.760, 1.000]
At least one non-philosopher	69	96	0.914	0.049	[0.798, 1.000]
Methods information					
Sample size reported	72	100	1	0.000	NaN^a^
Participant Demographics reported	72	100	1	0.000	NaN
Design reported	70	97	0.943	0.040	[0.843, 1.000]
Participant exclusions reported	69	96	0.915	0.048	[0.791, 1.000]
Descriptive statistics					
Central tendency measures (means, medians) and/or frequency measures (e.g., percentages)	71	99	0.850	0.147	[0.000, 1.000]^a^
Standard Errors (SE)	69	96	0.901	0.056	[0.771, 1.000]
Error bars in figures	71	99	0.978	0.022	[0.930, 1.000]
Inferential statistics					
NHST (*p*-values used)	71	99	0.793	0.201	[0.000, 1.000]^a^
Effect Size (ES) reported	71	99	0.972	0.028	[0.941, 1.000]
ES interpreted	71	99	0.966	0.034	[0.871, 1.000]
Confidence Intervals (CI) reported	71	99	0.965	0.035	[0.871, 1.000]
Prospective power reported	71	99	0.660	0.317	[0.000, 1.000]^a^
Observed power reported	69	96	0.707	0.159	[0.318, 0.933]^a^
Bayes Factor	72	100	NaN	NaN	NaN^a^

^a^At least one variable in the cross-tabulation is a constant

## Results

Table 2 shows a summary of descriptive results pertaining to the whole sample of papers.

**Table 2:** Summary of variables for all papers (*N* = 134)

Variable	*n*	% papers
Author characteristics		
First author a philosopher	95	71%
Corresponding author a philosopher	97	72%
At least one non-philosopher	73	54%
Methods information		
Sample size reported	133	99%
Participant Demographics reported	100	75%
Design reported	91	68%
M-Turk used for data collection	59	44%
Participant exclusions reported	59	44%
Descriptive statistics		
Central tendency measures (means, medians) and/or frequency measures (e.g., percentages)	130	97%
Standard Errors (SE)	37	28%
Error bars in figures	58	43%
Inferential statistics		
NHST (*p*-values) used	129	96%
Effect Size (ES) reported	71	53%
ES interpreted	29	22%
Confidence Intervals (CI) reported	38	28%
Prospective power reported	2	1%
Observed power reported	7	5%
Bayes Factor	2	1%

### Author Characteristics

Although over half of the papers had at least one non-philosopher co-author, the first and corresponding author of nearly three-quarters of them was a philosopher, suggesting that non-philosophers may often play an advisory role—perhaps on issues of methodology. This raises potential concerns about the large minority of papers that did not have co-authors with methodological and statistical expertise. We are, of course, unable to estimate the number of philosopher authors who had obtained relevant training, but we note again that statistics and experimental design are not typically part of a philosophical education. Of course, the mere fact that a co-author is not a philosopher does not automatically imply that they possess the relevant quantitative competence. We therefore checked the affiliations of the non-philosopher co-authors of papers in our sample: of the 73 papers that had at least one non-philosopher co-author, 59 had co-authors affiliated with Psychology or Cognitive Science, 7 with Economics or Business, 2 with Computer Science and 4 with medicine. Overall, the overwhelming majority of papers had a co-author from fields in which the acquisition of statistical and quantitative skills is a standard part of academic training.

### Methods Information

Details of methods were generally reported in reasonable detail. Nearly all the papers we examined reported sample size; a majority explicitly reported demographics, allowing for assessment of the study’s generalizability (at least with regard to age and gender, the most common reported categories). Most studies explicitly described the study’s design either in the relevant part of the Methods section or alongside the statistical test employed (we note that an explicit, formal description of the design—i.e., reporting whether the manipulation was within or between participants, specifying the independent and dependent variables, etc.—is not a universal requirement for scientific reports; although it is considered good practice, an attentive reader can usually fathom the design if the procedure is clearly described). A little under half of the studies were conducted online and data were collected using the M-Turk platform; this popular platform allows for efficient data collection, but has also been reported to yield discrepant results with laboratory studies in some experimental psychology paradigms (Crump et al. 2013), suggesting that its usefulness should not be taken for granted but rather evaluated on a case-by-case basis. Finally, fewer than half of the papers provided information about participants removed from analysis (both number of participants removed and reason for exclusion); it is impossible, of course, to know how many of the papers that did not report exclusions actually had any, but we note that exclusions—e.g., of outliers—are an extremely common practice in psychology.

### Descriptive Statistics

Nearly all the papers reported their results in terms of either central tendency measures (means, medians or modes) or frequencies (either raw numbers or percentages). Just over a quarter reported standard errors, although over 40% included error bars in figures. Of those papers whose figures showed error bars (*n* = 58), the bars represented either standard errors (*n* = 22, 38%) or confidence intervals (*n* = 17, 29%). One paper (~2%) included one figure showing standard errors and another figure showing confidence intervals, and the remaining papers showing error bars did not report what the bars represented in the figure(s) (*n* = 18, 31%). None of the papers that identified what their error bars represented used them to show standard deviations.

### Inferential Statistics

Almost all of the papers applied null hypothesis significance testing, but only just over half of them reported measures of effect size, and less than a quarter of the sample provided an interpretation of a reported effect size. Similarly, fewer than a third complemented the reported *p*-values with confidence intervals. A very small number of studies reported analyses of statistical power—either prospective or observed, despite a growing concern that the preponderance of underpowered studies is contributing to the high proportion of false positives in scientific publishing (Button et al. 2013; Open Science Collaboration 2015). Finally, only two studies employed Bayes Factors. Although this practice is not yet pervasive in related fields such as experimental psychology either, adopting Bayesian approaches has been recommended as a way to address the shortcomings of NHST.

### Association Between Number of Citations and Reporting Practices

Are better studies (or at least ones with better reporting) cited more? We performed point-biserial correlations to explore possible associations between number of citations and statistical reporting practices (specifically, whether or not ES, CI and SE were reported) as well as the association between number of citations and quality indicators of methodology reporting (whether or not descriptive statistics, sample size or demographics were reported).

We calculated correlations separately for each year in our sample, because number of citations is confounded by time since publication: naturally, the older studies had a higher mean number of citations (2013: *M* = 18.88, *SD* = 19.62; 2014: *M* = 20.86, *SD* = 30.97; 2015: *M* = 8.47, *SD* = 9.35; 2016: *M* = 4.57, *SD* = 5.10). For the newer studies (2015–16), not enough time has passed to assess with any reliability how well they have been cited.

Overall, our analysis indicates very little (if any) association between markers of reporting quality and citations: the majority of correlations were low and far from conventional statistical significance, and the few that reached significance were mostly negative (note that absence of a marker was always coded as ‘0’, and its presence as ‘1’, so a negative correlation suggests more citations when a marker was absent; this was the case in 2013 for reporting of demographics, and in 2014 for reporting effect size and design). Furthermore, none of the correlations replicated across years, and (considering the number of correlation analyses run) none would survive a correction for multiple comparisons (Table 3).

**Table 3:** Point-biserial correlations (r_pb_) between number of citations and methods information, descriptive statistics and inferential statistics by year

			r_pb_	*p*	Lower 95% CI	Upper 95% CI
Year 2013 (*n* = 41)						
Methods information						
Citations	–	Sample size	−0.091	0.573	−0.388	0.223
Citations	–	Demographics	−0.320 *	0.042	−0.571	−0.013
Citations	–	Design	−0.177	0.268	−0.460	0.138
Descriptive statistics						
Citations	–	Central tendency/Frequency	0.007	0.965	−0.301	0.314
Citations	–	Standard Errors (SE)	−0.190	0.235	−0.470	0.125
Inferential statistics						
Citations	–	Effect Size (ES)	−0.202	0.206	−0.480	0.113
Citations	–	Confidence Intervals (CI)	0.055	0.733	−0.257	0.357
Year 2014 (*n* = 22)						
Methods information						
Citations	–	Sample size	NaN^a^	NaN	NaN	NaN
Citations	–	Demographics	0.051	0.823	−0.379	0.462
Citations	–	Design	−0.515 *	0.014	−0.770	−0.120
Descriptive statistics						
Citations	–	Central tendency/Frequency	−0.017	0.940	−0.436	0.407
Citations	–	Standard Errors (SE)	−0.045	0.844	−0.458	0.384
Inferential statistics						
Citations	–	Effect Size (ES)	−0.437 *	0.042	−0.725	−0.019
Citations	–	Confidence Intervals (CI)	0.339	0.122	−0.096	0.666
Year 2015 (*n* = 34)						
Methods information						
Citations	–	Sample size	NaN ^a^	NaN	NaN	NaN
Citations	–	Demographics	0.247	0.160	−0.100	0.540
Citations	–	Design	0.362 *	0.035	0.027	0.624
Descriptive statistics						
Citations	–	Central tendency/Frequency	0.160	0.366	−0.188	0.473
Citations	–	Standard Errors (SE)	−0.013	0.941	−0.350	0.326
Inferential statistics						
Citations	–	Effect Size (ES)	−0.156	0.377	−0.470	0.192
Citations	–	Confidence Intervals (CI)	−0.051	0.774	−0.383	0.292
Year 2016 (*n* = 37)						
Methods information						
Citations	–	Sample size	NaN ^a^	NaN	NaN	NaN
Citations	–	Demographics	0.013	0.938	−0.312	0.336
Citations	–	Design	0.038	0.823	−0.290	0.358
Descriptive statistics						
Citations	–	Central tendency/Frequency	NaN ^b^	NaN	NaN	NaN
Citations	–	Standard Errors (SE)	−0.038	0.823	−0.358	0.290
Inferential statistics						
Citations	–	Effect Size (ES)	−0.018	0.915	−0.340	0.308
Citations	–	Confidence Intervals (CI)	−0.044	0.797	−0.363	0.284

*p < .05 (2-tailed)

** Bonferroni adj. α = .05/28, *p* < .0018; note that none of the correlations that reach conventional significance survive this correction

^a^ Variance = 0 for reporting of Sample size

^b^ Variance = 0 for reporting of Central tendency/Frequency

These findings indicate that there is no evidence that adopting better reporting practices is currently beneficial for authors in terms of getting cited. This may be due to a lack of awareness in the field: If authors are not aware of the need for such practices, they will not give adequate reporting appropriate weight in their assessment of a study’s value and their decision on whether to cite it, potentially leading to over-citation of methodologically flawed studies and under-citation of sound ones.

### Authors’ Disciplinary Background and Variables of Reporting Quality

Is having at least one non-philosopher among the authors associated with any of the reporting quality variables discussed above? We employed Chi-squares tests to explore the association between author composition (i.e., whether the author list included at least one non-philosopher vs all authors being philosophers) and various reporting measures. Reporting of CI was significantly associated with author composition (*χ* ^2^ (1) = 8.79, *p* = .003, φ = .256); the distribution of frequencies among these two variables is shown on Table 4. Interestingly, the table shows that papers written without the help of non-philosophers were more, not less, likely to report CIs. Neither of the remaining two tests for associations with reporting statistical measures yielded statistically significant findings (association with reporting ES: (*χ* ^2^ (1) < 1, *p* = .352, φ = .080; association with reporting SE: (*χ* ^2^ (1) = 1.22, *p* = .270, φ = .095).

**Table 4:** Frequencies for author composition by Confidence Intervals (CI) reporting

	CI		
At least one non-philosopher?	Not reported	Reported	Total
NO	36	25	61
YES	60	13	73
Total	96	38	134

Having at least one non-philosopher among the authors was also not associated with whether or not papers explicitly reported their design (*χ* ^2^ (1) < 1, *p* = .558, φ = .051), or demographics (*χ* ^2^ (1) < 1, *p* = .544, φ = .052).

### Testing Associations among Reporting-Quality Variables

We also explored possible associations between variables indexing statistical reporting-quality (reporting of ES and CI) and methods-reporting quality (reporting of design and demographics). A set of chi-square tests revealed that papers reporting effect sizes were also significantly more likely to report design details (*χ* ^2^ (1) = 13.16, *p* < .001, φ = .313) and demographics (*χ* ^2^ (1) = 19.20, *p* < .001, φ = .378; Tables 5 and 6 respectively). We found no significant association between reporting CI and design details (*χ* ^2^ (1) < 1, *p* = .368, φ = .078) or demographics (*χ* ^2^ (1) < 1, *p* = .470, φ = .062).

**Table 5:** Frequencies for effect size (ES) by reporting of design

	Design		
ES	Not reported	Reported	Total
Not reported	30	33	63
Reported	13	58	71
Total	43	91	134

**Table 6:** Frequencies for effect size (ES) by reporting of demographics

	Demographics		
ES	Not reported	Reported	Total
Not reported	27	36	63
Reported	7	64	71
Total	34	100	134

Finally**,** use of M-Turk was significantly associated with reporting exclusion of participants (*χ* ^2^ (1) = 6.06, *p* = .014, φ = .213, Table 7). We expand on the significance of this finding in the Discussion.

**Table 7:** Frequencies for use of M-Turk by exclusion of participants

	Participants excluded?		
M-Turk	No	Yes	Total
No	49	26	75
Yes	26	33	59
Total	75	59	134

## Discussion

Our analyses examined the reporting practices employed in a large sample of empirical studies published in leading philosophy journals over a recent 4-year period. We found that NHST (in the form of reporting *p*-values) is overwhelmingly the dominant statistical analysis approach. During the period we examined, the older field of experimental psychology has gradually acknowledged the shortcomings of over-reliance on *p*-values as a sole marker of findings’ meaningfulness, and reporting complementary measures such as effect sizes and confidence intervals has become common. In Experimental Philosophy, however, this is not yet the norm: only half of the papers we examined reported measures of effect size, and still fewer reported confidence intervals. (Admittedly, confidence intervals have a one-to-one relation with *p*-values, but they are widely viewed as being more straightforward to interpret).

Furthermore, it is now accepted in the fields of experimental psychology and cognitive neuroscience that underpowered studies have, in the past, led to an over-representation of false positives in the published record; this has led to a recent emphasis on using prospective power analysis, when possible, to pre-determine sample sizes; to a lesser extent, reporting of observed power has also increased. We find no evidence of this trend in the Experimental Philosophy literature: among the studies we assessed, a very small number made any reference at all to statistical power. Finally, very few studies employed more sophisticated statistical approaches, such as Bayes factor.

The results reported here suggest that to date, Experimental Philosophy has adopted analytical and reporting practices that are closer to those that dominated psychology and cognitive neuroscience before the re-examination prompted by recent concerns about a replication crisis (Button et al. 2013; Open Science Collaboration 2012, 2015). In our Introduction, we reviewed surveys of the Psychology literature that spanned the years 1996 to 2013. We showed that reporting of effect sizes, for example, has increased from 26% of the articles sampled in 1996–2000 (Matthews et al. 2008) to over 90% in a survey of articles published in Canadian psychology journals in 2013 (Counsell and Harlow 2017). The turning point seems to be after 2010, as a survey of papers from 2009 to 2010 still found effect sizes were reported in only about 40% of studies (Fritz et al. 2012); and a large-scale analysis of survey articles (Fritz et al. 2013) examining articles published in psychology journals between 1990 and 2010 found only 3% reported power analysis, 10% reported confidence intervals, and 38% reported effect sizes (although an upward trend across this period was noted for effect sizes). This has changed in recent years (though the process is still ongoing): Tressoldi et al. (2013), found that effect sizes and confidence intervals were reported in a majority of articles published in 2011 in both high and low impact journals (with the notable—and lamentable—exception of the highest-impact venues, Nature and Science), in some journals reaching 90% - the figure also found by Counsell and Harlow (2017). In light of this, our findings that only 53% of Experimental Philosophy articles in our sample reported effect sizes, and only 28% provided confidence intervals, suggest that statistical reporting practices in Experimental Philosophy seem to be lagging a few years behind those of comparable fields.

The studies we examined almost always provided information about sample size. Other important information about sample demographics and study design was less commonly (though frequently) reported. However, fewer than half of the studies directly referred to the number of participants that had been excluded from analysis. It is possible, of course, that the low proportion of reported exclusions is due to a low rate of exclusions in the studies themselves, and that all authors who excluded participants also reported this explicitly. However, it is noteworthy that participant exclusion is a highly-common practice in psychology and related fields; although there are often good justifications for doing so (e.g., when participants fail to engage with the task, are unable to perform it adequately, or have clear response biases), the practice has also been highlighted as an element of ‘researcher degrees of freedom’ (Simmons et al. 2011). Specifically, when exclusion criteria are not set a-priori (and reported as such), this leaves potential room for introduction of novel exclusion criteria after the results are known; this may, in turn, make it easier to obtain statistically-significant results—and due to the human susceptibility to cognitive biases, which even those who do research on such biases are not immune to (Simmons et al. 2011), the best researchers, armed with the best of intentions, may be unaware that they are using exclusion rules they would not have invoked before the data were known.

Our current sample gives reason to believe that participant exclusion may also be common in Experimental Philosophy, due to the large variety of criteria that have been applied when such exclusions were reported. On the one hand, as mentioned above, there are often perfectly valid reasons for excluding participants. On the other hand, however, the need to exclude a substantial number of participants (in some cases, over half) should be avoided as much as possible, to prevent concerns about researcher-degrees-of-freedom (Simmons et al. 2011) and statistical artefacts (Shanks 2017) as alternative explanations for reported findings. Several of the studies we surveyed excluded a large number of participants for failing basic comprehension tests or otherwise showing that they did not follow task requirements: For example, Wilkenfeld et al. (2016) tested 142 participants but also mention that a further 188 participants were excluded for failing to consent, failing to complete the experiment, or giving an incorrect response to one of the reading or comprehension questions; Horvath and Wiegmann (2016) excluded the data of 142 (out of 284) subjects who did not complete the survey or completed it in under 1 min; Berniūnas and Dranseika (2016) excluded 52 of 300 participants for failing a comprehension task; and Roberts et al. (2016) tested 140 participants but excluded 72 of them—65 for answering one or more comprehension questions incorrectly, and 7 because they had formal training in philosophy. When a large proportion of participants fails comprehension tests, this implies that the task design may have benefitted from additional piloting, prior to running the study, in order to make its content sufficiently clear to participants; and restrictions that disqualify from participation and can be known in advance (such as having formal training in philosophy) should be applied during initial participant screening rather than after data collection. The flipside of exclusion criteria is very strict inclusion criteria: Holtzman (2013) reported that out of 1195 participants recruited through blogs and social networks and who had completed his survey, he focused only on 234 philosophers who held a PhD or DPhil in philosophy. There is nothing wrong with conducting research on populations with specific educational or professional backgrounds; but ideally, recruitment procedures should prevent the sample from consisting mostly of participants who do not belong to the relevant population.

Most of the above examples are of studies that used online platforms for data collection. Although such platforms are incredibly useful, their use may also result in the recruitment of a high number of unsuitable participants or a low level of participant engagement, which can negatively impact the quality of the data collected. This attests to the difficulties involved in carrying out research online; however, such difficulties must be mitigated through rigorous recruitment procedures and the use of comprehensible tasks. Unless the measured variables are entirely independent of the exclusion criteria (a requirement that is very hard to verify), excessive post-hoc data selection—even when completely justified in light of the study’s goals—can lead to results that are pure artefacts resulting from regression to the mean (Shanks 2017). Finally, many of the concerns raised by data exclusion can be assuaged by adhering to two simple recommendations: Pre-registering the study before it is run, including details of its proposed exclusion criteria and analysis plans; and reporting the effect of exclusions on the results after the study is concluded. We go into further detail on both of these recommendations below.

The sample of studies covered by our analysis is representative of the work being published in leading philosophy journals, but is obviously not entirely comprehensive: some Experimental Philosophy articles have not been included in our sample because they were published in journals such as *Episteme*, an outlet that was not listed in the two rankings considered in this study. Furthermore, the sample of journals considered here is rather heterogeneous: for example, some of the journals that are classed here as philosophical, such as *Review of Philosophy and Psychology*, are outlets intended to attract genuinely interdisciplinary research. It should also be noted that the classification of authors as philosophers and non-philosophers is at least somewhat arbitrary. We considered the affiliation at the time of publication (usually given in the published article) but this might not fully capture the researcher’s educational background. Finally, it could be argued that the sample itself is not large enough, at 134 papers, to adequately cover the field’s norms on such a diverse range of variables, not all of which are relevant to all the papers in the sample. While we acknowledge that any sample meant to reflect a greater whole could benefit from being larger, we do believe that our principled choice of leading journals, combined with our methodology for selecting all the empirical papers these journals published over a substantial period, provides a representative picture of the state of the art as indicated by the field’s leading publication venues.

We also note that our coding strategy (a score of “0” for the answer “no”, and a score of “1” for the answer “yes”) has a limited resolution, meaning that items which varied in their degree of completeness could still be given the same score. Importantly, however, this is likely to have resulted in a more positive picture of reporting practices than the actual reality: Any mention of a relevant variable (e.g., effect size) would lead to a paper being assigned a value of 1 for that variable, even if the report itself was partial or applied inconsistently (or even incorrectly, an issue we did not delve into); a value of 0 was only assigned if the paper did not mention the variable at all. This may have somewhat inflated the number of papers coded with a value of 1 for any given variable.

On the other hand, the keyword-based search deployed here may have also occasionally missed some papers which did, in fact, report on a particular variable. In particular, in examining the reporting of study design features, we assessed whether the study was presented as “within subjects”, “between subjects”, “repeated measures” or “independent groups”; however, even in psychological research these labels are not universally used in reports; it is often assumed that educated readers would be able to infer such design features from the description of the study.

Notably, we focus here on the *type* of information reported, not on reporting or analysis errors. In the field of psychology, recent studies (Veldkamp et al. 2014; Nuijten et al. 2016) have focused instead on the prevalence of inconsistent *p*-values in top psychology journals by means of an automated procedure to retrieve and check errors in the reporting of statistical results. A recent application of this type of analysis to the field of Experimental Philosophy (Colombo et al. 2018) concludes that statistical inconsistencies are not more widespread in Experimental Philosophy than in psychology—meaning that when experimental philosophers use NHST, they do not tend to make consistency errors any more than psychologists do.

**Fig. 1:**
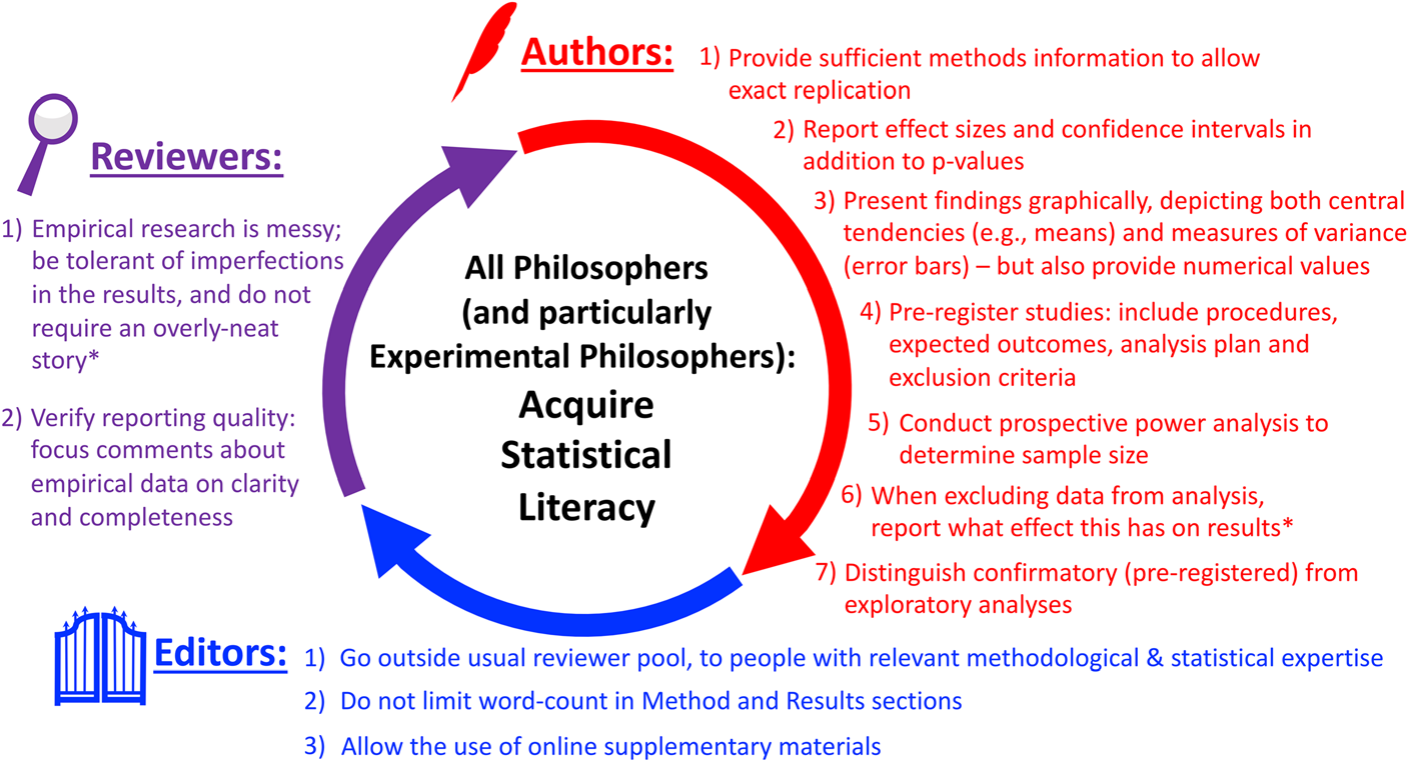
Recommendations for authors, editors and reviewers of Experimental Philosophy studies. This list complements the recommendations that Simmons et al. (2011) made for Psychology. We repeat two of their recommendations (marked with asterisks) but endorse all of their suggestions. The present recommendations build on practices that have been adopted in recent years in other empirical fields, but have yet to become the norm in Experimental Philosophy

Despite its limitations, we believe our study of current practices for reporting the design and analysis of Experimental Philosophy research offers interesting and potentially important findings. Such investigations provide insight into what researchers are doing well and what could be done to improve research and reporting practices in future studies. This complements direct assessments of replicability, such as the XPhi Replicability Project, a recent large-scale effort to reproduce central Experimental Philosophy findings (Cova et al. 2018https://osf.io/dvkpr/), which has provided encouraging data about current levels of replication in the field. We should not be complacent, though: Ensuring continued replicability requires the consistent adoption of appropriate reporting practices. We therefore end this report with a set of recommendations for authors, editors and reviewers of Experimental Philosophy papers (see Fig. 1 for a summary infographic).

We start with a general recommendation for philosophers and academic philosophy departments. A growing number of philosophers are carrying out empirical research, and an increasing number (in sub-fields such as philosophy of mind and philosophy of neuroscience and psychology) view empirical findings as directly relevant to their conceptual analysis. If this trend is to continue, it will become essential for philosophers to acquire statistical literacy as part of their education. Statistical analyses are the lens through which present-day science looks at empirical data. Therefore, an adequate understanding of statistics—including current developments and controversies in relevant fields—should not be outsourced to collaborators from other fields, but rather should become as integral to a philosopher’s education as courses in logic currently are.

As for authors, editors and reviewers, we strongly endorse the recommendations of Simmons et al. (2011), who made a list of suggestions aimed at reducing the number of false-positive publications by putting in place checks on experimenter degrees of freedom. These recommendations were aimed at researchers in psychology, but are equally applicable to any field in which statistics are used to analyze empirical data, and particularly to fields where those data are human behaviors, beliefs and attitudes. We will not repeat those recommendations here, but our recommendations below do include a couple of them that, in light of the present findings, seem to have particular relevance to Experimental Philosophy.

For example, it seems particularly necessary for authors in Experimental Philosophy to take heed of Simmons et al.’s (2011) recommendation that “If observations are eliminated, authors must also report what the statistical results are if those observations are included”. Further requirements also make sense in light of the large number of exclusions in some of the studies examined here (none of which report whether and to what extent application of exclusion or inclusion criteria affected the results): reports must commit to having defined the rules for exclusion prior to conducting any analysis (including the calculation of descriptive statistics), and must provide a clear rationale for such exclusions, to prevent ad-hoc removal of participants. Furthermore, to prevent undisclosed exclusions, papers should always explicitly report whether any participants were excluded or not.

More generally, transparency can be improved by adopting pre-registration. There is increasing support across the sciences for the idea of pre-registering studies, with initiatives such as the Preregistration Challenge (http://cos.io/prereg) offering assistance and incentives to conduct pre-registered research, and journals such as Psychological Science awarding ‘badges’ to papers that employ various good practices, including pre-registration. Current pre-registration platforms (e.g., the Open Science Framework, http://osf.io/; and AsPredicted, http://AsPredicted.org/) allow registration to consist simply of the basic study design, although they also enable inclusion of a detailed pre-specification of the study’s procedures, expected outcomes and plan for statistical analysis (including exclusion criteria). Importantly, pre-registering the analysis plan does not preclude analyses that were not originally considered, or further analyses on subsets of the data; rather, it enables a clear and transparent distinction between confirmatory (pre-registered) and exploratory analyses, with the acknowledgment that it is often the latter kind that leads to the most interesting follow-up research.

With regard to specific analysis techniques, NHST is the main approach to statistical analysis in Experimental Philosophy (and is still the norm in Experimental Psychology too). However, experimental philosophers should take heed of the recent move in psychology toward augmenting *p*-values with measures of effect size and increased use of confidence intervals. In particular, a paper’s discussion and interpretation of its findings should focus on effect sizes, as they are more informative than simply reporting whether a finding was statistically significant.

The use of other statistical approaches in place of NHST (e.g., Bayesian analysis) is also on the rise in psychology and other sciences, although the use of these approaches is still controversial: Simmons et al. (2011) oppose the adoption of Bayesian statistics as a way of addressing the shortcomings of *p*-values, noting that such analyses are prone to arbitrary assumptions (e.g., in the choice of prior probabilities) that, along with simply adding another set of tests to choose from, increase researcher degrees of freedom; several other authors (e.g., Dienes 2011, 2014; Kruschke 2013; Rouder et al. 2009), focus instead on the usefulness of Bayesian analyses for establishing whether the evidence supports the null hypothesis. Whatever the outcome of these debates, experimental philosophers should remain up to date on the current consensus regarding best practice.

Authors should also make sure they provide all the relevant information on both the methods and results. Although the vast majority of the studies we examined reported their sample size, a much smaller number reported sample demographics that would allow an assessment of their findings’ generalizability. Furthermore, many studies were vague on design and procedure details that determine whether a reader who wanted to conduct an exact replication would be able to do so. To facilitate clear and comprehensive writing, journal editors should recognize that word limits can be a serious obstacle to proper reporting of methods and results. In light of this, journals such as Psychological Science have now made clear that “The Method and Results sections of Research Articles do not count toward the total word count limit. The aim here is to allow authors to provide clear, complete, self-contained descriptions of their studies” (Psychological Science 2018). We suggest that editors of Philosophy journals should also consider revising their guidelines and strive to allow for sufficient level of detail in reporting.

Philosophers are not as accustomed as psychologists are to using graphs to make their point, but Experimental Philosophy authors should present their findings graphically if visualization allows for readers to better see trends and patterns (Matejka and Fitzmaurice 2017). For example, although there is some controversy about the use of bar-graphs to display results (see Bar Bar Plots Project 2017,5; Pastore et al. 2017), there is a consensus that bar graphs showing means are uninterpretable without including error bars representing standard errors, standard deviations, or confidence intervals; when including error bars, the measure they represent should be clearly indicated.

However, even when graphics are helpful, authors should always provide numerical values for descriptive statistics and effect sizes as well, so that the study can be included in future replication efforts, Bayesian analyses and meta-analyses. To avoid redundancy, numerical values that are represented in graphic depictions can be given in supplementary online information , which is allowed by most journals. In cases in which journals do not allow authors to use supplementary materials , editors and publishers should consider updating their editorial policies to allow for their use.

Further, it is the role of editors and reviewers to verify that appropriate reporting practices, including those detailed above, are adhered to. In particular, editors of philosophy journals that publish experimental papers should make it a habit to go outside their usual reviewer pool and seek reviewers with the relevant methodological and statistical expertise to evaluate the empirical aspects of the work.

Reviewers, for their part, should focus not only on the content of the findings but also make sure to address quality of reporting, verifying the clarity and completeness of empirical methods, and the use of statistical analyses that go further than simply reporting *p*-values. As recommended by Simmons et al. (2011), reviewers should also be tolerant of imperfections in the results—empirical data are messy, and an unrealistic expectation for perfectly neat stories is a strong incentive for researchers to apply so-called ‘researcher degrees of freedom’. Although we have no evidence that unrealistic demands are a particular problem amongst reviewers of Experimental Philosophy studies, we do note that real data often lend themselves less comfortably to the kind of air-tight conceptual arguments that philosophers are more accustomed to.

The rapid recent growth of Experimental Philosophy suggests exciting prospects for informing philosophical arguments using empirical data. This burgeoning field must, however, insure itself against facing its own replication crisis in years to come by taking advantage of insights reached, over the same recent period, by other fields; adopting best-practice standards in analysis and reporting should go a long way towards this goal.

## Footnotes

1
https://osf.io

2
https://www.psychologicalscience.org/publications/psychological_science/ps-submissions#STAT

3
https://scholar.google.co.uk/citations?view_op=top_venues&hl=it&vq=hum_philosophy

4 We used *Google Scholar* to count citations. Although this is not necessarily the most accurate database, it does incorporate citations from outside the field of philosophy (unlike Philpapers), and is not manually curated (unlike Scopus, Web of Knowledge or PubMed), meaning it offers the widest collection of citations for each of the papers we analysed.

5
https://www.kickstarter.com/projects/1474588473/barbarplots
